# Diabetes prevalence and associated risk factors among adults in rural Herat province, Afghanistan: a community-based cross-sectional study

**DOI:** 10.1186/s12889-026-27103-y

**Published:** 2026-03-24

**Authors:** Abdul Saboor Karim Suri, Khwaja Mir Islam Saeed, Said Naim Allemy

**Affiliations:** 1 Department of Curative Medicine, Faculty of Medicine, Jami University, Herat, Afghanistan; 2 Technical Advisor, Afghanistan National Public Health Institute, Ministry of Public Health, Kabul, Afghanistan

**Keywords:** Diabetes mellitus, Hypertension, Risk factors, Rural population, Non-communicable diseases, Afghanistan, WHO STEPS

## Abstract

**Background:**

Diabetes mellitus is a major public health concern in low- and middle-income countries, particularly in fragile health systems such as Afghanistan. Limited community-based evidence exists on the burden and correlates of diabetes among rural populations. This study aimed to estimate the prevalence of diabetes and identify its associated risk factors among adults living in rural areas of Herat province, Afghanistan, using the WHO Stepwise approach to noncommunicable disease (NCD) risk factor surveillance.

**Methods:**

A community-based cross-sectional survey was conducted from January to December 2024 among adults aged ≥ 18 years residing in rural areas of Herat province. A multistage cluster sampling technique was applied following the WHO STEPS methodology. Data were collected on sociodemographic characteristics, behavioral and metabolic risk factors using standardized questionnaires, physical measurements, and fasting blood samples. Diabetes was defined as fasting plasma glucose ≥ 126 mg/dL or self-reported use of antidiabetic medication. Both bivariable and multivariable logistic regression analyses were performed to determine factors associated with diabetes, with adjusted odds ratios (AOR) and 95% confidence intervals (CI) reported.

**Results:**

A total of 609 participants were included in the analysis (mean age: 40.6 ± 15.8 years; 51.9% were female). The overall prevalence of diabetes was 7.6% (95% CI, 5.6–9.9). In the multivariable analysis, age 45–54 years (AOR = 3.99; 95% CI: 1.31–12.16) and hypertension (AOR = 2.37; 95% CI: 1.29–4.36) were independently associated with diabetes. Education and central obesity were significant in bivariable analysis but lost significance after adjustment.

**Conclusions:**

The prevalence of diabetes among adults in rural Herat province is considerable, highlighting an urgent need for community-level screening and preventive interventions prioritizing older adults and individuals with hypertension. Strengthening primary health care and integrating NCD surveillance in rural Afghanistan are essential to reduce the growing burden of diabetes.

## Introduction

Diabetes mellitus (DM) represents a growing global health challenge, affecting populations in both high-income countries (HICs) and low- and middle-income countries (LMICs). While HICs face high morbidity rates, their robust health systems help mitigate the disease burden. In contrast, LMICs, particularly those with fragile health infrastructures like Afghanistan, face a disproportionate impact due to limited resources for management and prevention. According to the latest estimates from the International Diabetes Federation (IDF), approximately 537 million adults were living with diabetes in 2021, and this number is projected to rise to 783 million by 2045, with the largest relative increases expected in low- and middle-income countries (LMICs) [1, 2]. The condition contributes significantly to premature mortality, reduced quality of life, and increased healthcare expenditures worldwide [3].In South Asia, the prevalence of diabetes has reached alarming levels. For instance, a recent systematic review in Pakistan reported a diabetes prevalence ranging from 16% to 25% [4], and studies from Iran indicate similarly rising trends, with prevalence estimates exceeding 10% in many provinces [5]. These figures are driven by rapid urbanization, dietary changes, obesity, and physical inactivity [6–9].

Despite these global and regional concerns, Afghanistan remains among the least studied countries regarding the epidemiology of diabetes and related risk factors. The country faces multiple public health challenges, including limited resources, ongoing humanitarian crises, and fragile health infrastructure [10]. National surveillance systems for NCDs are still in the early stages of implementation, and population-based data, especially from rural areas, are scarce. Most available reports are limited to facility-based or small-scale studies conducted in urban settings, which do not reflect the true burden of diabetes across the broader Afghan population [11–13]. This lack of comprehensive data hampers the ability of policymakers to design and implement effective NCD prevention and control strategies.

The WHO STEPwise approach (STEPS) provides a standardized framework for monitoring key NCD risk factors, including diabetes, through community-based surveys [14]. This methodology enables countries with limited health information systems, such as Afghanistan, to generate nationally and regionally representative data for evidence-based policy-making [15]. Implementing the STEPS approach in rural Afghan communities is particularly critical because more than 70% of the population resides in rural areas, where access to health services and awareness of NCD prevention remain very limited [16, 17]. Moreover, rural residents may have distinct behavioral and metabolic risk profiles compared to urban populations due to differences in lifestyle, nutrition, and socioeconomic status [18].

Previous studies from neighboring countries, including Pakistan and Iran, have reported a rising prevalence of diabetes and its risk factors even among rural populations [19, 20]. However, similar large-scale community-based evidence from Afghanistan is almost non-existent. Understanding the prevalence and determinants of diabetes in these underserved rural communities is essential for developing cost-effective screening programs, allocating health resources, and integrating diabetes care into primary healthcare services, as recommended in the WHO Package of Essential NCD Interventions (PEN) for primary healthcare in low-resource settings [21].

While national surveillance efforts, such as the 2018 and 2024 Afghanistan STEPS surveys, provide valuable aggregate estimates showing a diabetes prevalence of approximately 11% [12, 13], they often mask the specific epidemiological profile of rural communities. Most existing subnational studies have focused on urban centers like Kabul [16] and Herat City [17], or are limited to specific northern rural districts like Andkhoy [18]. Currently, there is a lack of large-scale, community-based data specifically from the rural population of western Afghanistan in the post-2021 context. Therefore, this study was conducted to estimate the prevalence of diabetes and to identify its associated risk factors among adults aged 18 years and above living in rural areas of Herat province, Afghanistan, using the WHO STEPwise approach. By providing reliable community-based data, the study aims to fill a critical gap in national evidence and support policymakers in designing targeted interventions for rural settings.

## Methods

### Study design and setting

A community-based cross-sectional survey using the World Health Organization (WHO) STEPwise approach to noncommunicable disease (NCD) risk factor surveillance was conducted between January and December 2024 in rural areas of Herat province, western Afghanistan. Herat is one of the largest provinces of the country, with a predominantly rural population that relies mainly on agriculture and small-scale trading activities. The WHO STEPS methodology was chosen to ensure comparability with national and international NCD surveillance studies [14, 15].

### Study population and eligibility criteria

The target population consisted of adults aged 18 years and above who had been living in the selected rural communities of Herat province for at least six months. Individuals who were pregnant, critically ill, or unable to communicate during the interview were excluded.

### Sample size determination

The required sample size was calculated using the Cochran formula [22], assuming a 95% confidence level, 5% margin of error, and a design effect of 1.5, based on the most recent demographic estimates from the National Statistics and Information Authority (NSIA) of Afghanistan. An additional 10% was added to account for potential non-response. Accordingly, 643 individuals were initially selected. Of these, 24 samples were lost due to refusal to provide blood samples or laboratory errors, resulting in a final analytical sample of 609 participants.

### Sampling procedure

A multistage cluster sampling technique was applied in accordance with the WHO STEPS methodology [14]. Villages were first selected as primary sampling units using probability proportional to size, and households within each cluster were then selected systematically. One eligible adult from each household was randomly chosen for participation using the Kish method [23, 24].

## Data collection procedure

Data collection followed the three standardized STEPS:

- Step 1 (Questionnaire): Socio-demographic and behavioral data, including age, sex, education, occupation, tobacco use, diet, and physical activity, were collected using the standardized WHO STEPS questionnaire. This instrument is the officially nationally validated Dari version utilized in previous Afghanistan WHO STEPS surveys [12, 13], which had been culturally adapted for use in Afghanistan. Trained interviewers administered the questionnaire through face-to-face interviews after receiving three days of intensive training.
- Step 2 (Physical measurements): Anthropometric and blood pressure measurements were obtained using calibrated instruments. Height and weight were measured using a mechanical Frölix analog scale, and body mass index (BMI) was computed as weight (kg) divided by height squared (m²). Blood pressure was measured three times using a Yuwell aneroid sphygmomanometer (Two-Shelling model) after participants had rested for at least five minutes in a seated position, with 5-minute intervals between measurements. The average of the three readings was recorded.
- Step 3 (Biochemical measurements): Fasting blood samples were collected following at least eight hours of fasting. Serum glucose levels were analyzed in a certified laboratory using a Photometer 5010 biochemical analyzer (Germany).

### Definition of variables

Diabetes mellitus was defined as fasting plasma glucose (FPG) ≥ 126 mg/dL (7.0 mmol/L) or current use of antidiabetic medication, following WHO criteria [25].

Hypertension was defined as systolic blood pressure ≥ 140 mmHg and/or diastolic blood pressure ≥ 90 mmHg or current use of antihypertensive medication [26].

Overweight and obesity were classified according to WHO BMI categories: overweight (25.0–29.9 kg/m²) and obesity (≥ 30 kg/m²) [27].

Central obesity was defined as a waist circumference ≥ 94 cm for men and ≥ 80 cm for women [28].

Low vegetable intake was defined as consuming vegetables fewer than five days per week, based on WHO dietary recommendations [29].

Smoking was defined as current use of any tobacco product. Physical activity levels were classified according to WHO GPAQ recommendations; physical inactivity was defined as achieving less than 600 MET-minutes of activity per week. Regarding dietary fats, solid kitchen oil referred to hydrogenated vegetable oils or animal fats (ghee) solid at room temperature. Dyslipidemia components were defined based on NCEP ATP III guidelines: High Total Cholesterol (≥ 200 mg/dL), High LDL (≥ 130 mg/dL), Low HDL (< 40 mg/dL for men < 50 mg/dL for women), and High Triglycerides (≥ 150 mg/dL).

### Data analysis

Data were entered and analyzed using IBM SPSS Statistics version 26. Descriptive statistics (means, standard deviations, and proportions) were used to summarize participants’ characteristics. The prevalence of diabetes and 95% confidence intervals (CI) were calculated. Associations between diabetes and independent variables were examined using binary logistic regression. To prevent overfitting given the number of diabetes cases (*n* = 46) a parsimonious multivariable model was constructed [30]. Variables were selected based on statistical significance in bivariate analysis (*p* < 0.20) and biological plausibility (e.g., Age, Education) The ‘Job’ variable categories were merged (Employed vs. Unemployed/Housewife) to eliminate zero-cell counts. Adjusted odds ratios (AOR) and corresponding 95% CI were reported. Statistical significance was set at *p* < 0.05.

### Ethical considerations

The study was approved by the Institutional Review Board of the Ministry of Public Health of Afghanistan (Approval Code: A.1023.429). Written informed consent was obtained from all participants before data collection. Confidentiality and anonymity were strictly maintained throughout the study.

For participants identified as illiterate, the informed consent form was read verbatim in the local language (Dari) by a trained interviewer in the presence of a competent and impartial witness. Following the verbal affirmation of understanding by both the participant and the witness, the participant affixed their thumbprint to the consent form as documented proof of their voluntary participation.

## Results

### Sociodemographic characteristics and diabetes prevalence

A total of 609 participants were included in the final analysis. The mean age of the study population was 40.6 ± 15.8 years, and 51.9% (*n* = 316) were female. The majority of participants were illiterate (74.7%, *n* = 455) and married (95.1%, *n* = 579). Regarding employment, 42.2% (*n* = 257) were employed (official or physical labor), while 57.8% (*n* = 352) were unemployed or housewives.

The overall prevalence of diabetes mellitus in the study population was 7.6% (46/609; 95% CI: 5.6–9.9). As shown in Table 1, the prevalence of diabetes varied significantly across age groups (*p* < 0.001), rising from 2.5% in those under 35 years to 19.6% in the 45–54 age group. Participants with higher educational levels had a significantly lower prevalence of diabetes compared to illiterate participants (3.2% vs. 9.0%, *p* = 0.026). No statistically significant differences in diabetes prevalence were observed regarding gender, job category, monthly income, or marital status in the Bivariable analysis.

**Table 1:** Sociodemographic characteristics and their association with diabetes mellitus among adults in rural Herat, Afghanistan (*N* = 609)

Variable	Categories	Total *N*	Diabetic *n* (Row %) [95% CI]	Crude OR (95% CI)	*P*-value
Age Group (years)	< 35	275	7 (2.5) [1.0–5.1]	Ref	< 0.001
	35–44	117	7 (6.0) [2.4–11.9]	2.44 (0.84–7.11)	0.103
	45–54	107	21 (19.6) [12.6–28.4]	9.35 (3.84–22.75)	< 0.001
	≥ 55	110	11 (10.0) [5.1–17.2]	4.25 (1.60–11.28)	0.004
Gender	Male	293	21 (7.2) [4.5–10.8]	Ref	0.743
	Female	316	25 (7.9) [5.2–11.4]	1.11 (0.61–2.03)	
Education Level	Illiterate	455	41 (9.0) [6.5–12.0]	Ref	0.026
	Literate	154	5 (3.2) [1.1–7.4]	0.34 (0.13–0.87)	
Job Category	Unemployed/Housewife	352	27 (7.7) [5.1–11.0]	Ref	0.892
	Employed	257	19 (7.4) [4.5–11.3]	0.96 (0.52–1.76)	
Monthly Income	< 10,000 AFN	108	10 (9.3) [4.5–16.4]	Ref	0.485
	> 10,000 AFN	163	19 (11.7) [7.2–17.6]	1.29 (0.58–2.87)	
Marital Status	Married	579	45 (7.8) [5.7–10.2]	Ref	0.366
	Unmarried	29	1 (3.4) [0.1–17.8]	0.42 (0.06–3.19)	

*CI* Confidence Interval, *OR* Odds Ratio, *AFN* Afghani currency

Behavioral and Metabolic Risk Factors Table 2 presents the behavioral risk factors associated with diabetes. Sedentary behavior (reclining or sitting for ≥ 3 h per day) was the only behavioral factor significantly associated with diabetes in the Bivariable analysis (*p* = 0.031). The prevalence of diabetes was 10.2% among those with sedentary lifestyles compared to 5.5% in the more active group (Crude OR = 1.96; 95% CI: 1.06–3.60). Other behavioral factors, including smoking, snuff use, fruit and vegetable consumption, type of cooking oil, and physical activity levels, did not show statistically significant associations with diabetes.

**Table 2:** Bivariable analysis of behavioral risk factors for diabetes

Variable	Categories	Total *N*	Diabetic *n* (Row %) [95% CI]	Crude OR (95% CI)	*P*-value
Smoking	No	567	43 (7.6) [5.6–10.1]	Ref	0.916
	Yes	42	3 (7.1) [1.5–19.5]	0.94 (0.28–3.15)	
Mouth Snuff	No	467	31 (6.6) [4.5–9.3]	Ref	0.124
	Yes	142	15 (10.6) [6.0–16.9]	1.66 (0.87–3.17)	
Fruit/Veg Intake	≥ 3 days/week	110	8 (7.3) [3.2–13.8]	Ref	0.907
	< 3 days/week	499	38 (7.6) [5.4–10.3]	1.05 (0.48–2.32)	
Kitchen Oil	Liquid	32	6 (18.8) [7.2–36.4]	Ref	0.106
	Solid	546	39 (7.1) [5.1–9.7]	0.33 (0.13–0.88)	
	Both	31	1 (3.2) [0.1–16.7]	0.14 (0.02–1.28)	
Sedentary Time	< 3 h/day	345	19 (5.5) [3.4–8.5]	Ref	0.031
	≥ 3 h/day	264	27 (10.2) [6.9–14.5]	1.96 (1.06–3.60)	

While solid oil showed a protective OR in this specific categorization, the association was not statistically significant in the overall test (*p* = 0.106), likely due to the skewed distribution of oil types

Regarding metabolic risk factors (Table 3), general obesity (BMI ≥ 30 kg/m²) and central obesity were significantly associated with diabetes (*p* = 0.033 and *p* = 0.020, respectively). Specifically, participants with central obesity had twice the odds of having diabetes compared to those with normal waist circumference (Crude OR = 2.05; 95% CI: 1.12–3.75). Furthermore, hypertension showed a strong association with diabetes; the prevalence of diabetes was significantly higher among hypertensive individuals (12.7%) compared to normotensive individuals (5.5%) (Crude OR = 2.50; 95% CI: 1.36–4.60, *p* = 0.003). Lipid profile parameters did not show significant associations with diabetes in this population.

**Table 3:** Bivariable analysis of metabolic risk factors

Variable	Categories	Total *N*	Diabetic *n* (Row %) [95% CI]	Crude OR (95% CI)	*P*-value
General Obesity	Non-obese	557	40 (7.2) [5.2–9.7]	Ref	0.033
	Obese (BMI ≥ 30)	52	6 (11.5) [4.4–23.4]	1.69 (0.68–4.19)	
Central Obesity	No	377	21 (5.6) [3.5–8.4]	Ref	0.020
	Yes	232	25 (10.8) [7.1–15.6]	2.05 (1.12–3.75)	
Hypertension	No	436	24 (5.5) [3.6–8.1]	Ref	0.003
	Yes	173	22 (12.7) [8.1–18.7]	2.50 (1.36–4.60)	
Total Cholesterol	< 190 mg/dL	571	41 (7.2) [5.2–9.6]	Ref	0.187
	≥ 190 mg/dL	38	5 (13.2) [4.4–28.1]	1.96 (0.73–5.29)	

### Multivariable analysis

To identify independent predictors of diabetes, a multivariable logistic regression model was performed. Variables with statistical significance or conceptual importance (Age, Gender, Education, Job, Central Obesity, and Hypertension) were entered into the model. As detailed in Table 4, after adjusting for potential confounders, older age and hypertension remained independently associated with diabetes. Participants aged 45–54 years had approximately 4 times higher odds of diabetes compared to those under 35 years (Adjusted OR = 3.99; 95% CI: 1.31–12.16, *p* = 0.015). Similarly, individuals with hypertension had 2.37 times higher odds of having diabetes compared to those without hypertension (Adjusted OR = 2.37; 95% CI: 1.29–4.36, *p* = 0.006). Education and central obesity, which were significant in the Bivariable analysis, did not retain statistical significance in the adjusted model.

**Table 4:** Multivariable logistic regression analysis of factors associated with diabetes mellitus

Variable	Category	Adjusted OR (95% CI)	*P*-value
Age Group	< 35	Ref	-
	35–44	1.63 (0.50–5.28)	0.415
	45–54	3.99 (1.31–12.16)	0.015
	≥ 55	2.11 (0.71–6.25)	0.177
Gender	Male	Ref	-
	Female	0.69 (0.33–1.46)	0.334
Education	Illiterate	Ref	-
	Literate	0.66 (0.22–2.03)	0.477
Job	Unemployed/Housewife	Ref	-
	Employed	0.72 (0.32–1.62)	0.428
Central Obesity	No	Ref	-
	Yes	1.34 (0.64–2.80)	0.443
Hypertension	No	Ref	-
	Yes	2.37 (1.29–4.36)	0.006

Variables included in the model: Age, Gender, Education, Job, Central Obesity, and Hypertension

## Discussion

### Summary of key findings

In this community-based cross-sectional study, the prevalence of diabetes mellitus (DM) was found to be 7.6% among adults in rural Herat. In the bivariable analyses, several factors such as older age, lower educational attainment, central obesity, sedentary behavior, and hypertension were significantly associated with diabetes. After adjustment for potential confounders in the multivariable logistic regression model, older age and hypertension were identified as independent factors associated with diabetes.

### Comparison with national, regional, and global evidence

The prevalence identified in this study is lower than national estimates. The 2024 Afghanistan STEPS-based survey reported diabetes in 11.1% of adults and impaired fasting glucose in 10.3% [13], while a meta-analysis estimated a pooled national prevalence of approximately 12% [14], confirming the ongoing epidemiological transition toward higher NCD burdens [15]. Subnational studies also demonstrate variability: 13.2% in Kabul [16], 9.9% in urban Herat [17], and 9.7% in rural Andkhoy [18]. Our estimate is consistent with these rural findings and supports the urban–rural gradient, whereby urbanization, sedentary behavior, and dietary westernization increase diabetes risk.

Regionally, diabetes prevalence remains substantially higher in South Asia. A 2024 systematic review from Pakistan found prevalence ranging from 16% to 25% [4], and a 2025 meta-analysis estimated 19.1% for diabetes and 14.4% for prediabetes across 42 studies [29]. In India, the 2023 ICMR–INDIAB survey reported prevalence between 9% and 20% across states [31]. A 2025 regional synthesis further indicated that most South Asian nations now exceed 10–15% adult prevalence [5]. Globally, the International Diabetes Federation (IDF) estimated a 10.5% adult prevalence in 2021, projected to rise to 12.2% by 2045, with the steepest increases expected in low- and middle-income countries [2]. Although rural Afghanistan currently shows lower prevalence, this trend is unlikely to persist without active prevention and early detection strategies.

### Interpretation of determinants

Interpretation of Associated Factors Hypertension and older age emerged as the strongest independent correlates of diabetes in our final model. The strong association between hypertension and diabetes (AOR = 2.37) aligns with global evidence linking insulin resistance to endothelial dysfunction and vascular stiffness [32]. Similarly, the progressive risk observed with increasing age reflects the cumulative impact of metabolic exposure and beta-cell senescence [22]. Interestingly, central obesity and education, which were significant in bivariable analysis, did not retain significance in the multivariable model. This suggests a “suppression effect” or statistical collinearity, where the effect of adiposity might be mediated through hypertension or age pathways, a phenomenon observed in other South Asian datasets [32–34]. Regarding behavioral factors, our study highlighted two concerning trends. First, sedentary behavior (≥ 3 h of reclining/sitting per day) was associated with a twofold increase in diabetes risk in the unadjusted analysis. Second, the consumption of fruits and vegetables was alarmingly low (mean < 3 days/week). Although these dietary factors did not reach statistical significance in the multivariable model likely due to the homogeneous nature of the diet in rural settings their public health importance remains critical, as sedentary lifestyles and poor diet are well-established precursors to metabolic syndrome [35–37].

### Possible explanations for observed differences

The relatively lower prevalence in rural Herat compared to urban centers may reflect higher physical activity levels related to agricultural labor and lower exposure to processed westernized diets. However, underdiagnosis remains probable. Although we utilized laboratory-based fasting plasma glucose (FPG) analysis, reliance on FPG alone can miss cases that HbA1c or oral glucose tolerance tests (OGTT) would detect [38]. Limited diagnostic capacity and lack of routine screening in rural areas contribute to potential underestimation of the true burden.

### Policy and public health implications

Integrating diabetes management into Afghanistan’s broader NCD control framework is essential. The clustering of metabolic and socioeconomic risk factors highlights the need for community-based, multi-sectoral interventions. Community Health Workers (CHWs) a cornerstone of Afghanistan’s primary health system can play a vital role in screening, early referral, and lifestyle education. Evidence from neighboring South Asian countries demonstrates that CHW-led lifestyle interventions can effectively reduce diabetes risk and are cost-efficient in low-resource contexts [5].

At the policy level, institutionalizing routine WHO STEPS surveillance with biochemical components is critical to monitor national and provincial trends [25]. Nutrition-sensitive policies, including local vegetable promotion, subsidies for healthy foods, and restriction of trans fats, should be prioritized [37]. Cross-border collaboration with Pakistan and India can strengthen regional strategies for diabetes prevention. Aligning national health initiatives with Sustainable Development Goal (SDG) 3.4 which calls for a one-third reduction in premature NCD mortality by 2030 offers a global framework for action [39].

### Strengths and limitations

This study is among the few population-based investigations of diabetes in rural Afghanistan. Strengths include probability-based cluster sampling, adherence to the standardized WHO STEPS protocol, and the use of laboratory-confirmed fasting glucose. The inclusion of behavioral, socioeconomic, and clinical variables enhanced internal validity and provided a multidimensional assessment of risk.

However, the study has limitations. First, the cross-sectional design limits causal inference. Second, regarding outcome ascertainment, diabetes was defined based on a single Fasting Plasma Glucose (FPG) measurement using a biochemical analyzer. While this ensures analytical accuracy, clinical guidelines typically recommend repeat testing on a separate day for confirmation. Similarly, hypertension was defined based on the mean of three blood pressure readings taken during a single visit. Although this follows the WHO STEPS surveillance protocol for low-resource settings, it differs from clinical diagnostic criteria which require elevated readings on multiple occasions. Consequently, this ‘single-visit’ approach might have slightly overestimated or underestimated the true prevalence compared to a clinical setting. Finally, the exclusion of urban participants limits generalizability to the broader population, and self-reported data may have introduced recall bias.

Despite these limitations, the study provides valuable baseline evidence to guide national diabetes surveillance and prevention efforts.

## Conclusion

In summary, older age and hypertension were identified as independent factors associated with diabetes in this rural Afghan population, while central obesity and education showed significant associations only in unadjusted analyses. These findings illustrate the intertwined burden of vascular and metabolic disorders and emphasize the need for community-based, integrated hypertension and diabetes screening programs, particularly targeting older adults and those with elevated blood pressure in rural Afghanistan.

## Data Availability

The datasets generated and/or analyzed during the current study are available from the corresponding author upon reasonable request.
